# Feasibility of a multifaceted intervention to improve treatment initiation among patients diagnosed with TB using Xpert MTB/RIF testing in Uganda

**DOI:** 10.1371/journal.pone.0265035

**Published:** 2022-06-17

**Authors:** Stella Zawedde-Muyanja, Joseph Musaazi, Barbara Castelnuovo, Adithya Cattamanchi, Achilles Katamba, Yukari C. Manabe

**Affiliations:** 1 The Infectious Diseases Institute, College of Health Sciences, Makerere University, Kampala, Uganda; 2 Division of Pulmonary and Critical Care Medicine and Center for Tuberculosis, University of California San Francisco, San Francisco, California, United States of America; 3 Department of Medicine, School of Medicine, Makerere University College of Health Sciences, Kampala, Uganda; 4 Division of Infectious Diseases, Department of Medicine, Johns Hopkins University School of Medicine, Baltimore, Maryland, United States of America; Indian Institute of Technology Delhi, INDIA

## Abstract

**Background:**

One in five patients diagnosed with TB in Uganda are not initiated on TB treatment within two weeks of diagnosis. We evaluated a multifaceted intervention for improving TB treatment initiation among patients diagnosed with TB using Xpert^®^ MTB/RIF testing in Uganda.

**Methods:**

This was a pre-post interventional study at one tertiary referral hospital. The intervention was informed by the COM-B model and included; i) medical education sessions to improve healthcare worker knowledge about the magnitude and consequences of pretreatment loss to follow-up; ii) modified laboratory request forms to improve recording of patient contact information; and iii) re-designed workflow processes to improve timeliness of sputum testing and results dissemination. TB diagnostic process and outcome data were collected and compared from the period before (June to August 2019) and after (October to December 2019) intervention initiation.

**Results:**

In September 2019, four CME sessions were held at the hospital and were attended by 58 healthcare workers. During the study period, 1242 patients were evaluated by Xpert^®^ MTB/RIF testing at the hospital (679 pre and 557 post intervention). Median turnaround time for sputum test results improved from 12 hours (IQR 4–46) in the pre-intervention period to 4 hours (IQR 3–6) in the post-intervention period. The proportion of patients started on treatment within two weeks of diagnosis improved from 59% (40/68) to 89% (49/55) (difference 30%, 95% CI 14%-43%, p<0.01) while the proportion of patients receiving a same-day diagnosis increased from 7.4% (5/68) to 25% (14/55) (difference 17.6%, 95% CI 3.9%-32.7%, p<0.01).

**Conclusion:**

The multifaceted intervention was feasible and resulted in a higher proportion of patients initiating TB treatment within two weeks of diagnosis.

## Introduction

Despite being both preventable and curable, tuberculosis (TB) remains a major cause of morbidity and mortality globally. In 2019, there were 10 million incident cases of TB, 1.2 million deaths among HIV-negative people and an additional 200,000 deaths among patients co-infected with HIV [1]. Despite having 16% of the global population, sub-Saharan Africa reported 25% of global TB cases and more than half of all deaths [1]. In order to accelerate progress towards the sustainable development goal (SDG) targets to reduce TB deaths by 90% and decrease TB incidence by 80% over the next ten years, access to high-quality TB care services must be improved.

Universal access to high-quality TB care services is a key component of the End TB Strategy; TB care services should be of sufficient quality to improve the health of patients and free of catastrophic costs [2, 3]. However, in high TB and TBHIV burden countries, geographical, economic and socio-cultural barriers to accessing TB care services are considerable [4, 5] and result in about 30% of incident TB cases not initiating TB treatment annually [6]. This gap between incident and notified cases is high in the sub-Saharan African region where weak healthcare systems manifested by poor infrastructure and insufficient numbers of trained healthcare workers result in both underdiagnosis and underreporting of diagnosed TB cases [4, 7, 8]

In Uganda, improvements in screening practices and in the availability of TB diagnostics have led to an increase in the number of patients being diagnosed with TB annually. However, one in five patients diagnosed with TB experiences pretreatment loss to follow-up (are not initiated on TB treatment within two weeks of diagnosis) [9–12]. These are often HIV-positive patients who seek care at large tertiary referral hospitals [13]. In the six months following TB diagnosis, patients who experience pretreatment loss to follow-up are three times more likely to die than those initiated on TB treatment [14]. In our previous work, we identified health facility level barriers including: lack of awareness about the magnitude of pretreatment loss to follow-up (LFU) among both clinical and laboratory staff, difficulty in accessing sputum test results for both requesting clinicians and patients due to long turnaround times and inability to trace patients due to poor recording of patient locators. Patient level barriers included insufficient TB knowledge and lack of transport fares to make return journeys to the health facilities for treatment initiation [15]. We developed a multifaceted intervention to address these barriers and sought to test its feasibility and effectiveness for improving TB treatment initiation at a large tertiary referral hospital in Uganda.

## Methods

### Study design and setting

This was a pre-post interventional study at one tertiary referral hospital in eastern Uganda (Jinja regional referral hospital) which serves about two million people from five districts. The hospital offers TB diagnostic and treatment services and uses Xpert^®^ MTB/RIF testing as the initial diagnostic test for patients who present with signs and symptoms of TB. The hospital also extends Xpert^®^ MTB/RIF testing services to 25 primary care facilities within a 20–30 km radius through a diagnostic hub system where sputum samples are brought by motorcycle to the diagnostic hubs and results are returned to the primary care facilities by the same means. TB treatment is offered on an outpatient basis, unless there is an indication for hospital admission, and is free of cost to the patients. During a previous formative study to assess magnitude and factors associated with pretreatment loss to follow-up [13], this hospital had the highest proportion of patients experiencing pretreatment loss to follow-up with up to 30% of all patients diagnosed with TB not being initiated on TB treatment within two weeks of diagnosis.

### Theoretical model

We previously carried out a qualitative study to understand barriers to and facilitators of TB treatment initiation [15]. This study utilized the Capacity, Opportunity, Motivation and Behavior (COM-B) model for its theoretical framework. The COM-B model recognizes that behavior is the result of an interacting system involving Capacity -the necessary knowledge and skills; Opportunity—the social or environmental factors and Motivation–the reflective and autonomic processes that guide behavior [16, 17]. The model further acknowledges that changing behavior involves influencing one or more of the above components and identifies nine intervention functions outlined in its Behavior Change Wheel (BCW) that can be applied to achieve this. These intervention functions include persuasion, education, restriction, environmental restructuring, modelling, enablement, coercion and incentivization [16]. The model has been used to develop implementation strategies for cancer screening guidelines in Tanzania [18], to develop intervention strategies to improve diabetes medication adherence in South Africa [19] and gas stove use in Guatemala [20]. Our intervention strategy targeted the following barriers identified during the qualitative evaluation: a) reduced capacity of healthcare workers to put in place interventions to improve linkage to TB treatment due lack of knowledge about the problem b) reduced opportunity to communicate patient results and conduct home-based patient follow-up visits due to poor recording of patients’ contact information and c) reduced opportunity for patients to initiate TB treatment due to long turnaround times for sputum test results.

### Interventions

Our study utilized three intervention functions from the BCW. The first intervention was education, defined as increasing knowledge or understanding about a behavior [21]. We provided information on the magnitude of pretreatment loss to follow-up at the hospital and on the consequences of pretreatment loss to follow-up including increased mortality. This information was provided to the healthcare workers during continuous medical education sessions held at the hospital at the beginning of the intervention. We hypothesized that healthcare workers would be more willing to engage in other interventions to reduce pretreatment loss to follow-up if they had an increased understanding of the problem. Next, we restructured the work environment to increase the physical opportunity to initiate patients on TB treatment. We placed modified laboratory request forms at all TB screening points to improve recording of patient locators and increase the likelihood of successfully tracing patients who did not initiate TB treatment. These forms had additional fields e.g. the name and contact of village council leader- if the patient had no phone number- and increased space to record details of the patients’ residence (S1 Fig). Forms were carbonized so that copies of laboratory requests could be retained at the clinics. In addition, we reorganized the workflow to improve laboratory results turnaround time (TAT). In the OPD, batched delivery of sputum samples at the end of each work day was replaced with periodic deliveries throughout the day. In the laboratory, we replaced batched analysis of sputum samples with on-delivery analysis. Finally, we enabled quick communication of Xpert^®^ MTB/RIF positive results from the laboratory to the clinic staff and from the clinic staff to the patients by providing desk phones to both the laboratory and the outpatient department.

### Outcomes

The primary outcome was the proportion of patients diagnosed with TB who were initiated on TB treatment within two weeks of diagnosis. A secondary outcome was the proportion of patients with confirmed TB diagnosed and treated on the same day as they presented to the clinic (same-day diagnosis and treatment).

### Intervention evaluation

We evaluated the impact of the multifaceted intervention both quantitatively and qualitatively.

### Quantitative data

Data were collected on each of the key intervention components including: the number of healthcare workers who attended the medical education sessions, the proportion of patients for whom adequate locator data (name, age, village and phone number) was collected and laboratory turnaround time (time between sample receipt and results dispatch).

Data were collected on linkage to TB treatment including the number of patients started on TB treatment within two weeks of diagnosis and the proportion who received same day treatment initiation. We compared these data for the three months period before (June to August 2019) and after (October -December 2019) the intervention.

### Qualitative data

Qualitative data was collected through a focus group discussion conducted at the hospital by SZM one of the study investigators. The purpose of the focus group discussion was to understand healthcare workers’ lived experiences with the different intervention components. The discussion explored three main areas a) which aspects of the intervention were implemented well; b) challenges that were faced and c) what could be improved. Healthcare workers working in the outpatient clinic, the HIV clinic and the laboratory were invited to participate in the focus group discussion at the end of the workday. Participants’ responses were recorded using both hand written field notes and audio recording.

### Data analyses

Participants’ characteristics at pre- and post-intervention were presented using frequencies and percentages and compared using independent Person chi-squared test. We described two of the key intervention functions: a) recording of patient locators and b) sputum turn-around time pre- and post-intervention using proportions with 95% confidence intervals (CIs) estimated using normal approximation to binomial methods. Unadjusted estimates for the primary and secondary outcomes were described using frequencies and percentages. Differences in pre- and post-intervention proportions were compared using the independent Pearson chi-squared test. Because the outcome was common (>10%), we obtained adjusted estimates (predicted probabilities) for the primary outcome by fitting a Poisson regression model (with robust standard errors) and adjusting for sex, age groups, care entry point, HIV status and distance to the health facility. Subgroup analyses were performed to examine if the impact of intervention varied across participant characteristics using Poisson regression models (with robust standard errors) and fitting interactions between patient characteristics and the intervention period. Further, we generated piece-wise linear regression models to examine underlying secular trends within the pre-and post-intervention periods. Significance tests throughout the analyses was at 5% level. Analysis was conducted using Stata, version 14 (Stata Corporation, College Station, TX, USA).

Qualitative data from the focus group discussion were transcribed and coded by a study investigator (SZM) and another independent coder who had training in social sciences and experience with qualitative research. A deductive approach was used to generate themes along the three main areas of inquiry. Emerging themes were illustrated using participant quotes. All analysis was done using NVivo 12 software.

### Ethics statement

This study was approved by the Makerere University School of Medicine Research and Ethics Committee of the College of Health Sciences (Ref: 2016–132) and by the Uganda National Council of Science and Technology. Data on GeneXpert testing and TB treatment initiation was collected as part of routine care and was analyzed anonymously. Administrative clearance to conduct this analysis was obtained from the hospital administration. Written informed consent was obtained from healthcare workers who took part in the focus group discussion. Permission was sought from the respondents before audio recording the interview.

## Results

### Patient characteristics

Altogether, 1236 patients were evaluated for TB at the hospital; 679 (54.9%) before the intervention and 557 (45.1%) after the intervention. The majority of the patients evaluated for TB were female and 60% were from the outpatient clinic (Table 1).

**Table 1:** Baseline characteristics of patients.

Characteristic	Pre-intervention N = 679	Post intervention N = 557	P value
**Sex**			
Male	300 (44.2)	241 (43.3)	0.75
Female	379 (55.8)	316 (56.7)	
**Age**			
<15	62 (9.1)	23 (4.1)	<0.01
15–24	91 (13.4)	108 (19.4)	
25–34	128 (18.8)	113(20.3)	
35–44	169 (24.9)	113 (20.3)	
45–54	109 (16.0)	102 (18.3)	
>55	120 (17.7)	98 (17.6)	
**Care-entry point**			
Outpatient Clinic	341 (50.2)	402 (72.2)	<0.01
HIV Care Clinic	124 (18.3)	57 (10.2)	
Inpatient ward	209 (30.8)	82 (14.7)	
Antenatal Clinic	5 (0.7)	16 (2.9)	
**HIV status**			
Positive	153 (22.5)	131 (23.5)	<0.01
Negative	157 (23.1)	173 (31.1)	
Unknown	369 (54.4)	253 (45.4)	
**Distance to health facility**			
≤ 5 km	188 (27.6)	156 (28.0)	0.58
> 5km	253 (37.3)	194 (34.8)	
Unknown	238 (35.1)	207 (37.2)	

### Implementation of key intervention functions

#### Healthcare worker education

Fifty-eight healthcare workers including; 10 clinical officers, seven laboratory staff, 25 nurses, 16 lay health providers (HIV linkage facilitators and community healthcare workers) attended four CME sessions held at the outpatient clinic, the HIV clinic, the TB clinic and in the laboratory. The magnitude of pretreatment loss to follow-up at the hospital, mortality outcomes of patients who were not initiated on TB treatment plus suggested interventions to reduce pretreatment loss to follow-up were presented and discussed.

#### Reporting of patient locators

The proportion of patients who had a phone number recorded in the presumptive TB register or laboratory register improved from 46.1% (313/679) before the intervention to 60.8% (339/557) after the intervention (difference 14.7%, 95% CI 9.2%–20.3%, p<0.01). Although there was a slight decline in the proportion of patients whose residence was accurately recorded from (71% (482/679) before the intervention to 67.2% (374/557) during the intervention, this decrease was not statistically significant (difference -3.8%, 95% CI -9.0%—+1.3%, p = 0.09) (Table 2).

**Table 2:** Percentage differences in key intervention functions pre and post intervention.

Variable	Pre-Intervention	Post-Intervention	Percentage difference, post–pre (95% CI)	P-value^†^
**Key intervention functions**				
Number of participants	***679***	***557***		
*Patient Locator Recording*				
Had village address recorded, n (%)	*482(71*.*0)*	*374(67*.*2)*	-3.8 (-9.0 to 1.3)	0.09
Had phone number recorded, n (%)	*313(46*.*1)*	*339(60*.*8)*	14.7 (9.2 to 20.3)	<0.01
*Sputum Results turn-around time (hours)* *				
Median turn-around time, hours (IQR)	*14(4–47)*	*4 (3–6)*		
*Categories*, *n (%)*				
< 12	*331(48*.*7)*	*548(97*.*3)*	48.6 (44.6 to 52.6)	<0.01
12–24	*86(12*.*7)*	*11(2*.*0)*	-10.7 (-13.5 to -8.0)	
>24	*262(38*.*6)*	*4(0*.*7)*	-37.9 (-41.6 to -34.1)	

95%CI for the prevalence estimates obtained using normal approximation to binomial methods.

^†^ all p-values were obtained using independent Pearson chi-square test. All analysis significance test is at 5% level

* Sputum turn-around time is time from receipt of sputum samples in the lab to availing sputum test results to clinician

#### Restructuring of health facility workflow

During the intervention, the proportion of sputum samples analyzed within 12 hours of being received in the lab improved from 48.7% (331/679) to 97.3% (548/557) (difference 48.6%, 95% CI 44.6%-52.6%, p<0.01). The median turnaround time for all sputum samples improved from 14 (IQR 4–47) hours before the intervention to 4 (IQR 3–6) hours during the intervention (Table 2).

### Impact of intervention on key study outcomes

#### Primary outcome

The proportion of bacteriologically confirmed TB patients initiated on TB treatment within two weeks of diagnosis improved from 58.8% (40/68) before the intervention to 89.1% (49/55) after the intervention (difference 30.3%, 95% CI 16.0%–44.6%, p<0.01). After adjusting for participants’ characteristics (sex, age groups, care entry point, and distance to health facility), the proportion of patients initiated on TB treatment within two weeks was 65.8% before the intervention and 86.8% after the intervention (difference 20.9% 95% CI 3.2 to 38.7, p = 0.02) (Table 3).

**Table 3:** Percentage differences in study outcomes pre and post intervention.

Variable	Pre-Intervention	Post-Intervention	Percentage difference, post–pre (95% CI)	P-value^†^
Key Study Outcomes	N = 68	N = 55		
*Primary Outcome*				
Initiated within 14 days (primary outcome)	40 (58.8)	49 (89.1)	30.3 (16.0 to 44.6)	<0.01
*Secondary Outcome*				
Median time to TB treatment initiation (days -IQR)	*5(2–9)*	*1(0–2)*		
Initiated on treatment on the same day	5(7.3)	15 (27.3)	20 (6.6 to 33.2)	<0.01
**Adjusted analysis for primary outcome** ^¶^				
Initiated TB treatment within 14 days since TB diagnosis, %	65.8	86.8	20.9 (3.2 to 38.7)	0.02

95%CI for the prevalence estimates obtained using normal approximation to binomial methods, except for the adjusted analysis

^†^Except for the adjusted analysis, all p-values were obtained using independent Pearson chi-square test. All analysis significance test is at 5% level

^††^ Participants who were not initiated on TB treatment: 23/68 (34%) at pre-intervention and 5/55 (9%) at post-intervention

^¶^ Adjusted estimates obtained using Poisson regression with robust standard errors adjusting for: sex, age groups, care entry point and distance from home to health facility.

#### Secondary outcome

Among those treated, the median time to treatment initiation improved from 5 (IQR 2–9 days) to 1 (IQR 0–2 days) after the intervention. The proportion of patients who received same-day diagnosis and treatment improved from 7.4% (5/68) to 27.3% (14/35) after the intervention (difference 17.6%, 95% CI 6.6%–33.2%, p<0.01). (Table 3).

#### Subgroup analysis

**Fig 1:**
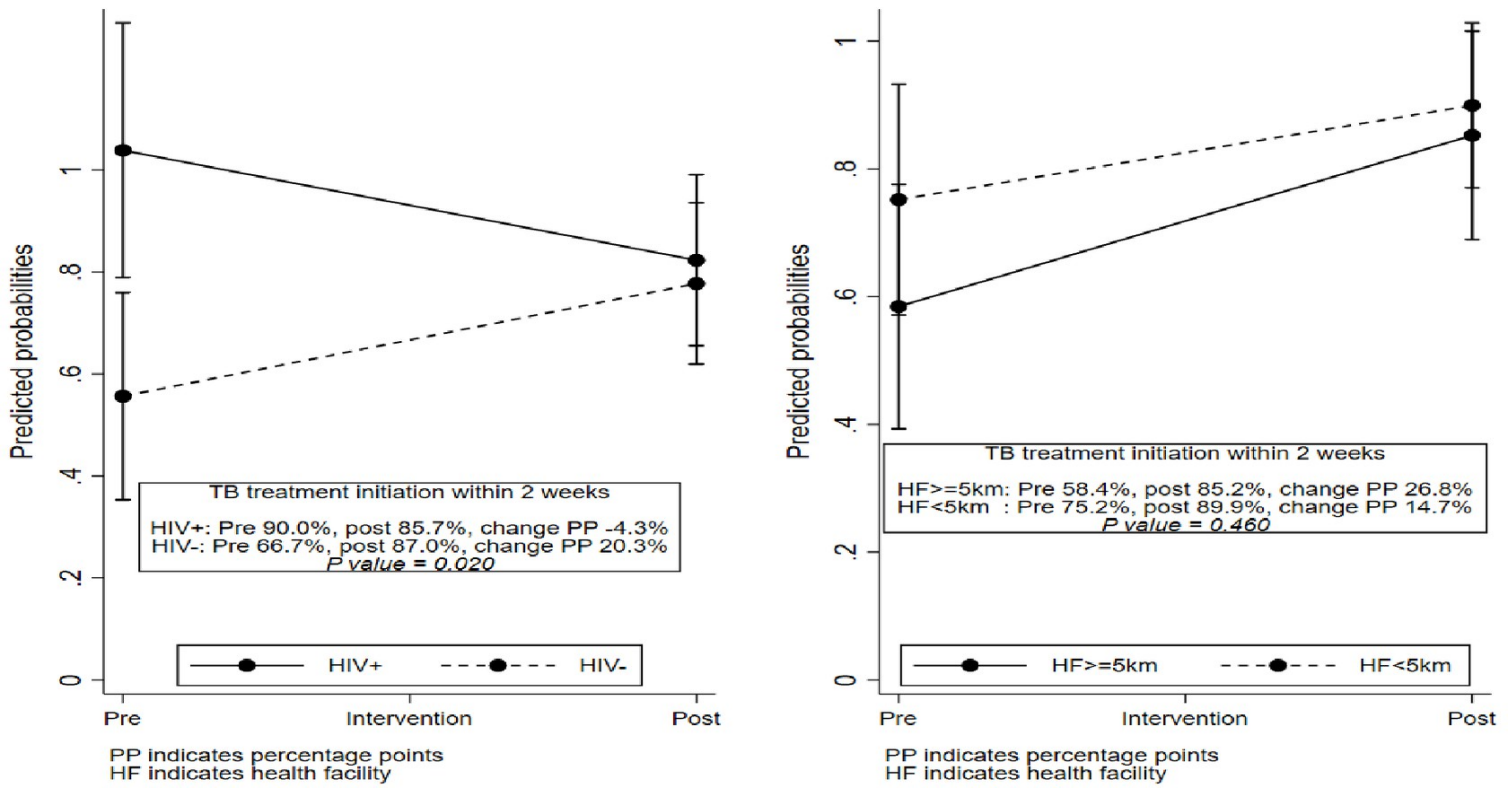
Subgroup analysis: Predicted probabilities of TB treatment initiation within two weeks by HIV status and distance to the health facility. Probabilities predicted after fitting a Poisson regression model (with robust standard errors) with interaction between intervention period and a patient characteristic, adjusting for other patient characteristics: sex, age groups, care entry point, distance to health facility and HIV status.

Subgroup analysis across patients’ characteristics showed that the increase in the proportion of patients initiated on TB treatment within two weeks of diagnosis was higher among HIV negative than HIV positive patients (HIV positive: 90.0% to 85.7%, difference -4.3% versus HIV negative: 66.7% to 87.0%, difference 20.3%; p-value 0.02) (Fig 1). The increase in the proportion of patients initiated on TB treatment within two weeks of diagnosis was also higher among patients who lived >5 kms from the health facility than among those who lived ≤5 kms from health facilities (≤5 kms: 58.4% to 85.2%, difference 26.8% versus > 5km: 75.2% to 89.9%, difference 14.7%), however the difference did not reach statistical significance (p-value 0.46) (Fig 1). The differences in proportion of patients started on TB treatment within two weeks of diagnosis were similar across sex, age groups and care entry points (p-values >0.05 for all comparisons). There were no secular trends in the pre- and post-intervention periods for the primary outcome (test for trend p-value 0.92 for pre-intervention period and 0.88 for post-intervention period).

#### Reasons for not initiating TB treatment

Reasons for not initiating TB treatment within two weeks of diagnosis in the post-intervention period included: death prior to treatment initiation (2 patients); declining TB treatment (1 patient) and travel outside the district for work (3 patients– 1 eventually initiated treatment after three weeks).

#### Results of qualitative evaluation

Eight healthcare workers participated in the discussion including five nurses, two laboratory healthcare workers and one counsellor. During the focus group discussion, healthcare workers confirmed that improved documentation of patient locators, reduced laboratory TAT and improved communication of sputum test results were key to improving TB treatment initiation at this hospital. However, they noted that documentation of patient locators particularly villages of residence could be further improved (Table 4). Healthcare workers also noted that same-day diagnosis did not always result in same-day treatment initiation due to factors beyond their control e.g. closure of the TB clinic after hours and over the weekend. In some cases, treatment initiation was delayed despite early communication of test results because patients lacked resources to come back to the hospital. Table 4 provides a summary of healthcare workers’ evaluation of the intervention.

**Table 4:** Key themes emerging from qualitative evaluation.

**What worked well**	
**Theme**	***Illustrative Quotes***
Intervention resulted in improved documentation of patient contacts.	*“We became serious with getting telephone numbers from clients*. *That one helped us to see that we get to our clients in time; for those ones who had telephone number and also for those one without telephone numbers*, *at least we were getting telephone number of relatives and at least we had very few patients who got lost”*. **[Nurse, Outpatient Clinic]**
	*“Samples were being delivered on time from the OPD and on top of that the request form would have all the data about the patient so if you get a positive*, *then you know who to call whether the outpatient clinic or the ART clinic”*. **[Lab Technologist]**
During the intervention, batched analysis of sputum samples was decreased.	*“In the lab*, *we were able to handle patients’ samples the way they come in*. *That really worked so well for us to be able to release results on time*, *to have the same day results”*. **[Lab Technologist 1]**
Intervention resulted in improved communication of sputum test results	*“Whenever results were being released*, *depending on the results*, *we were able to contact the clinician*, *just only to ensure our patients are linked on treatment on time*. *The availability of airtime on the phones really contributed towards the achievement of that”*. **[Lab Technologist 2]**
	*“But the communication was enabling us and even the availability of airtime to call patients to come back for treatment*, *did a very good work*, *because you may get results but I may not have airtime on my phone to call the patient*. *But since the airtime was available*, *it was easy to call the patients and they come that one helped us to link the patients on treatment* **[Nursing Assistant, Outpatient Clinic]**
**What did not work well**	
**Theme**	***Illustrative Quotes***
Staffing remained a challenge particularly in the laboratory.	*There were some staff changes*, *MH was taken away which created a challenge*, *because AL was mainly in administrative work*, *we then started not getting results well …we got another volunteer FL*. *FL helped us do good work*. **[Head, Outpatient Clinic]**
Occasionally, there was delayed treatment initiation even when a same-day diagnosis was made.	*For patients who came like around midday*, *results are ready the same day but by the time the results are out*, *those patients have already gone home*. *So*, *you try to call the client*, *will say that*, *“I have already gone*.*” Then you try to call you say*, *“You come back*,*” they say” no*, *I will not be able because of transport; I will come another day”*. **[Healthcare worker, Outpatient Clinic]**
Limited operating hours for TB treatment.	*We also had a hindrance of patients coming in over the weekend*. *Yes*, *you know weekends some units [the TB clinic] don’t work over the weekend*. *So*, *we could get some positive patients*, *then you call wards [to see if they can start the patient on TB treatment]*, *but they were not able to link them that very day to treatment*. **[Lab Technologist 1]**
	*Then*, *other challenges of the weekend patients*. *You know*, *this is more of the staffing issues*, *like on the TB ward we are few*. *So*, *you find most of the weeks*, *we don’t have staff on weekend*. *And yet patients still come in on the weekend*. *Yaah that was beyond our capacity but because it was staffing issues*, *we cannot solve*. **[Healthcare worker -TB Clinic]**
TB Stigma.	*Writing down patient’s phone numbers helped us to reduce our initial loss to follow-up because for each*
	*patient we were getting in the lab*, *we were able to trace*. *Apart from those ones*, *who refused*, *saying ah*..*I don’t have TB*. *Like the other lady who just refused to come for treatment*. **[Healthcare worker -TB Clinic]**
**What can be improved?**	
**Theme**	***Illustrative Quotes***
Documentation of patient locators.	*There was still that gap*, *some patients do not have complete documentation which gives the lab people challenges to trace which care-entry points these patients come from*. *This makes our work not good at all because you don’t where the patient is from*, *you don’t know who to call*. *This affects our same-day linkage*. *By the time you get the patient*, *it is three or four days down the road*. **[Lab Technologist 2]**

## Discussion

Although the WHO has clear guidelines for treatment initiation for patients diagnosed with TB, pretreatment LFU is a persistent problem in high TB burden settings. Several studies have described this problem [8, 11, 22–24] but fewer studies have evaluated interventions to address it. Our study examined the feasibility and preliminary effectiveness of a multifaceted intervention developed using the COM-B model. During the three months that this intervention was implemented, there was an improvement in both the proportion of patients initiating TB treatment within two weeks of diagnosis and in the time to TB treatment for patients presenting at the hospital with signs and symptoms of TB.

The components of our intervention have been previously studied. Continuous medical education has been shown to be effective in promoting the delivery of public health interventions [25]. Provision of continuous medical education can enhance knowledge, skill and self-efficacy of healthcare workers to engage in healthcare provision. However, continuous medical education has been shown to be more effective when combined with other interventions such as clinical support and mentorship [26], performance review and feedback [27] and restructuring of the work environment and lab strengthening [28–30].

In our study, we combined continuous medical education with restructuring of the work environment and enablement to enhance the physical opportunity for healthcare workers to initiate patients on TB treatment. These interventions reinforced the knowledge gained by healthcare workers and contributed to improving TB treatment initiation. For example, on-demand analysis of sputum samples decreased turnaround time while provision of desk phones enabled immediate communication of results to both healthcare workers and patients. Similar improvements in treatment initiation have been demonstrated following reductions in laboratory turnaround times for sputum test results. At primary healthcare facilities in Uganda, daily transportation of sputum samples to central laboratories (GeneXpert hubs) followed by prompt relay of sputum test results through text messaging resulted in a 20% increase in the proportion of patients initiating TB treatment [31]. In South Africa, Xpert^®^ MTB/RIF placement at the point of care rather than in a central laboratory decreased laboratory turnaround time and resulted in significant improvements in both the proportion of patients starting TB treatment and the time to TB treatment initiation [32, 33].

Our intervention resulted in an increase in the proportion of patients who initiated TB treatment at their initial clinic visit (same-day diagnosis and treatment initiation). Same-day diagnosis and treatment initiation is recommended by the WHO particularly in settings where patients are likely to default from the TB diagnostic pathway [34]. However, the WHO also acknowledges that significant organizational changes are required to achieve this. Our study demonstrates the kind of organizational changes required to achieve same-day diagnosis and treatment initiation and further highlights additional barriers e.g., lack of transport fares to return to health facility and limited operating hours for the TB clinic, that need to be overcome in order to ensure that a higher proportion of patients are initiated on TB treatment on the same day that they present to the health facilities. Finally, future interventions to improve TB treatment initiation should consider additional interventions e.g. the use of additional point-of-care diagnostics e.g. urinary LAM which could further reduce laboratory turnaround time; including transport reimbursements for patients initiating TB treatment and extending operational hours during which TB treatment can be initiated either through integration with other inpatient services or provision additional staff for the TB clinic.

Death prior to TB treatment initiation -signifying delayed presentation to care and TB stigma were some of the causes of pretreatment LFU in our study. Although early presentation followed by prompt treatment initiation remain top global priorities for achieving favorable treatment outcomes, delayed presentation remains prevalent in high TB burden resource limited settings [35–37]. Delays as long as 8–10 weeks between symptom onset and care seeking have been previously reported in Uganda and are associated with low TB knowledge, low socio-economic status and TB stigma [38, 39]. TB stigma caused by misconceptions about disease transmission or the association of TB with other stigmatized conditions e.g. HIV and poverty is a significant barrier to care seeking and treatment uptake [40, 41]. Interventions to improve TB knowledge, promote early care seeking and reduce TB stigma should be carried out at community level.

Our study had several strengths. First, our intervention was developed based on a theoretical framework, following a series of formative studies. This systematic development ensures that interventions match elicited barriers and is recommended by the UK Medical Research Council’s (MRC) guidance for developing complex interventions [42]. Secondly, our intervention targeted multiple barriers making it more likely to be effective [43]. Third, our outcome measures were objective and we measured intermediate process outcomes such as laboratory turnaround time which made our study outcomes more attributable to the intervention implemented. Finally, our work was done in a large public hospital which increases the generalizability of our findings to similar settings in high TB burden, low resource settings.

Our study also had several weaknesses. First, this was a quasi-experimental study. Therefore, the observed improvement in TB treatment initiation may attributed to other temporally related changes such as reduced patient loads. To address this, we collected data at multiple time points (S1 Table) and checked for underlying secular trends across both study periods. We observed no secular trends in the primary outcome pre and post intervention. Secondly, the observed improvements could be attributed in part to healthcare worker awareness that they were being observed (Hawthorne effect). This was minimized by not collecting data during the first month of the intervention. Despite these limitations, the study shows the potential impact of our multifaceted intervention to reduce pretreatment LFU and highlights areas that could be further improved to achieve earlier treatment initiation among patients diagnosed with TB.

## Conclusion

Pretreatment LFU remains a weak point in the TB care cascade that must be improved in order to achieve the WHO END TB goals. Successful interventions to improve TB treatment initiation have the potential to improve the population level impact of Xpert^®^ MTB/RIF and result in a significant decrease in TB incidence and mortality over time [44].

We found that a multifaceted intervention used existing diagnostic tools but focused on systems improvement had the potential to improve TB treatment initiation at a large tertiary facility in Uganda. Further evaluation of this intervention through a randomized trial is needed to prove its impact in similar public health facilities.

## Supporting information

S1 Fig Modified GeneXpert request and result form. (TIF)

S1 Table Trend of key intervention functions: Pre and post intervention. (TIF)

S1 Data (CSV)
