# Supplementary figures and images for: Feasibility of a multifaceted intervention to improve treatment initiation among patients diagnosed with TB using Xpert MTB/RIF testing in Uganda
Source: PLoS One. 2022 Jun 17;17(6):e0265035. doi: 10.1371/journal.pone.0265035 (PMC9491700; doi:10.1371/journal.pone.0265035)

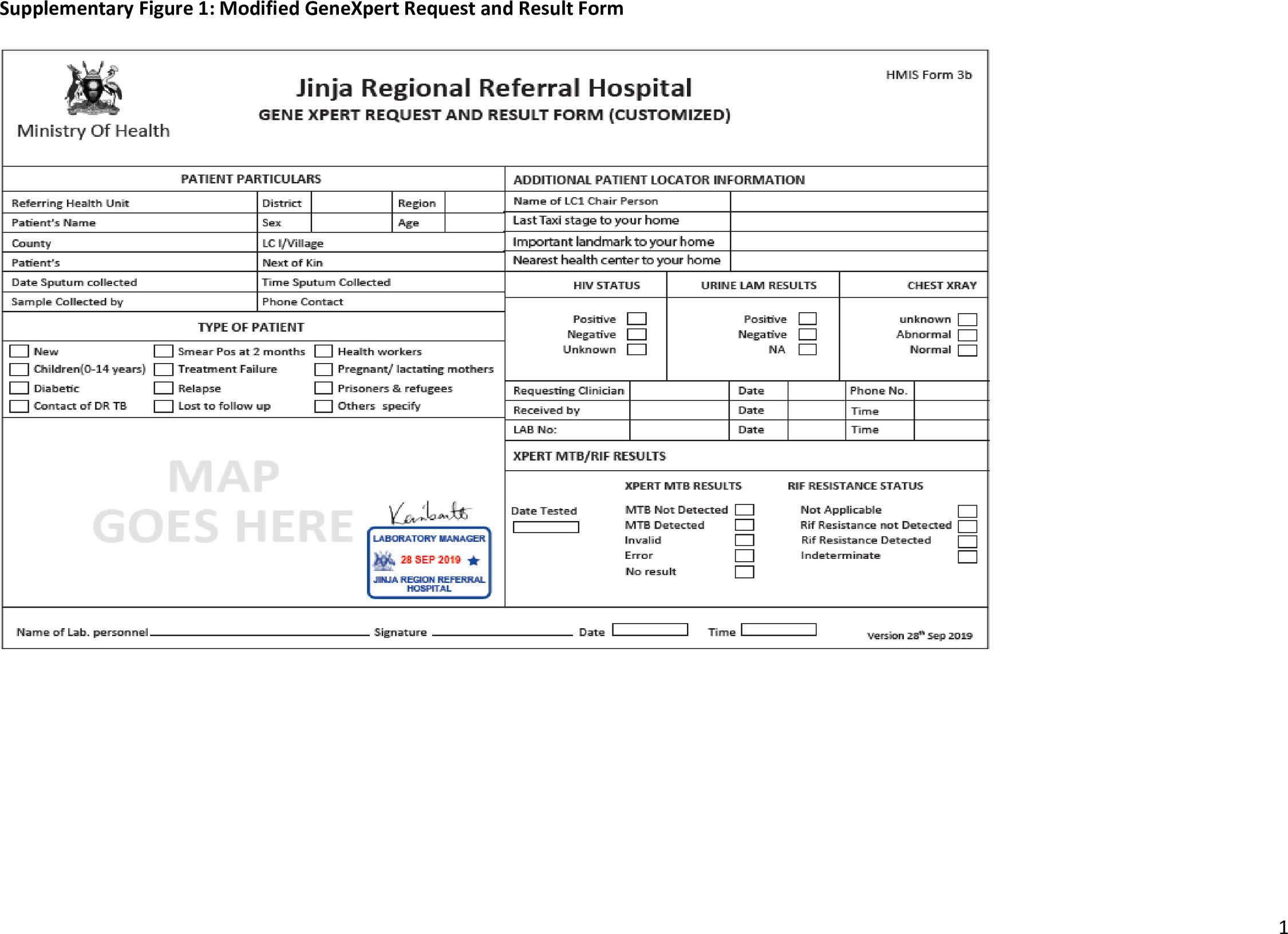

Supplement: S1 Fig — (TIF) [file pone.0265035.s001.tif]

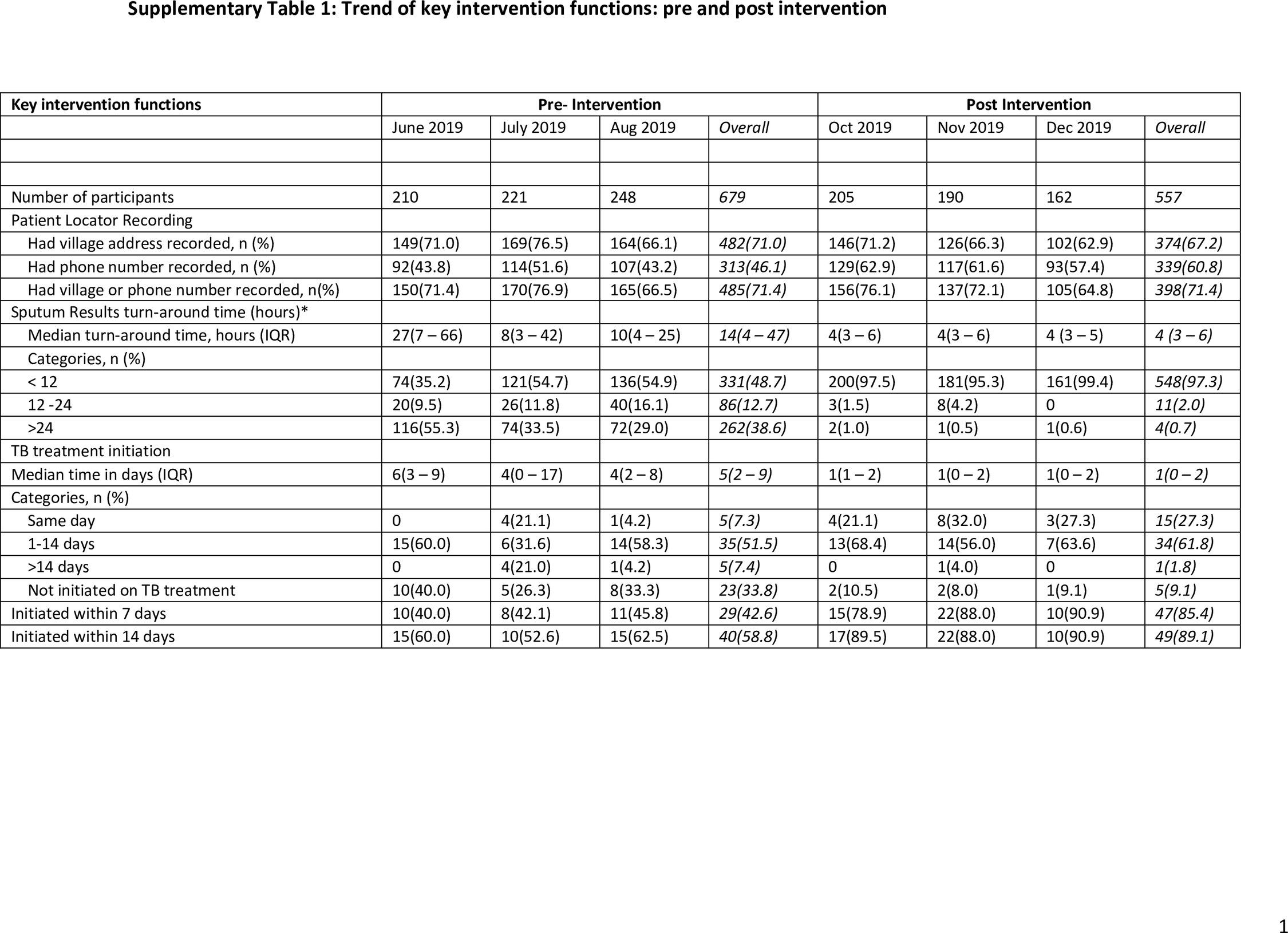

Supplement: S1 Table — (TIF) [file pone.0265035.s002.tif]
